# Alcohol Use, Working Conditions, Job Benefits, and the Legacy of the “Dop” System among Farm Workers in the Western Cape Province, South Africa: Hope Despite High Levels of Risky Drinking

**DOI:** 10.3390/ijerph110707406

**Published:** 2014-07-21

**Authors:** J. Phillip Gossage, Cudore L. Snell, Charles D. H. Parry, Anna-Susan Marais, Ronel Barnard, Marlene de Vries, Jason Blankenship, Soraya Seedat, Julie M. Hasken, Philip A. May

**Affiliations:** 1 Center on Alcoholism, Substance Abuse and Addictions, The University of New Mexico, Albuquerque, NM 87106, USA; E-Mails: jgossage@unm.edu (J.P.G.); jblanken@unm.edu (J.B.); 2 School of Social Work, Howard University, Washington, DC 20059, USA; E-Mail: csnell@howard.edu; 3 Alcohol, Tobacco and Other Drug Research Unit, Medical Research Council of South Africa, Tygerberg 7505, South Africa; E-Mail: charles.parry@mrc.ac.za; 4 Department of Psychiatry, Stellenbosch University, Tygerberg 7505, South Africa; E-Mails: asmarais@sun.ac.za (A.-S.M.); ronelb07@gmail.com (R.B.); mmdevries@sun.ac.za (M.M.V.); sseedat@sun.ac.za (S.S.); 5 Nutrition Research Institute, Gillings School of Global Public Health, The University of North Carolina at Chapel Hill, Kannapolis, NC 28081, USA; E-Mail: jhasken@email.unc.edu

**Keywords:** alcohol use and abuse, epidemiology, fetal alcohol spectrum disorders (FASD), farm workers, South Africa

## Abstract

This study describes alcohol consumption in five Western Cape Province communities. Cross-sectional data from a community household sample (n = 591) describe the alcohol use patterns of adult males and females, and farm workers *vs.* others. Data reveal that men were more likely to be current drinkers than women, 75.1% *vs.* 65.8% (*p* = 0.033); farm laborers were more likely to be current drinkers than individuals in other occupations 83.1% *vs.* 66.8% (*p* = 0.004). Group, binge drinking on weekends was the norm; men were more likely to be binge drinkers in the past week than women 59.8% *vs.* 48.8% (*p* = 0.086); farm workers were more likely to binge than others 75.0% *vs.* 47.5% (*p* < 0.001). The legacy of “Dop” contributes to current risky drinking behaviors. Farm owners or managers were interviewed on 11 farms, they described working conditions on their farms and how the legacy of “Dop” is reflected in the current use of alcohol by their workers. “Dop” was given to farm workers in the past on six of the 11 farms, but was discontinued for different reasons. There is zero tolerance for coming to work intoxicated; farm owners encourage responsible use of alcohol and assist farm workers in getting help for alcohol problems when necessary. The farm owners report some positive initiatives, were ahead of the movement to provide meaningful wages, and provide other important amenities. Further research is needed to assess whether progressive practices on some farms will reduce harmful alcohol use.

## 1. Introduction

Alcohol consumption has been a mainstay of farm life is South Africa (SA) since Dutch settlers began colonizing the country in the 17th century. As a condition or benefit of employment, farm workers were provided food and wine, instead of wages as partial payment for labor. In some farms wine was reported to have been provided five times a day [1]. This practice of payment was known as the “Dop” system [2,3,4,5,6,7]. A “dop” is the Afrikaans word for “tot”. Giving wine as remuneration became illegal in 1961, but a loophole in the law allowed alcohol provisions to continue as a gratuity and/or reward [1]. Cultural practices of regular drinking over the last 300 years is believed to be manifest in risky or harmful drinking in certain substrata of SA society. Binge drinking in our research is five or more drinks per occasion for men, and three or more drinks for women; chronic alcohol consumption at binge levels can lead to adverse (physical or mental) health outcomes. In this paper, the terms “risky”, “harmful”, “hazardous” or “problematic” refer to those drinking behaviors that may result in adverse health outcomes, injury or death [8,9,10,11]. The consequences include: social disruption, lost worker productivity, diversion of financial resources from necessities to the purchase of alcohol, injury, domestic violence, child neglect, and the malady that has been the focus of our research team since 1996, fetal alcohol syndrome (FAS). FAS is currently known as part of a continuum and referred to as fetal alcohol spectrum disorders (FASD). FASD can occur when a woman consumes risky levels of alcohol during a pregnancy [12]. The Western Cape Province (WCP) has some of the highest rates of FASD in the world [13,14,15,16,17,18,19,20,21,22,23,24].

The primary goal of this paper was to link this historical perspective with more recent information and survey data on the harmful use of alcohol by farm workers (see Brumby *et al.* [25] for a discussion of alcohol use in Australian farming communities). Our focus was to learn directly from some citizens and farm owners/managers in our catchment area about the current drinking practices and legacy of Dop on their farms. Is there progress being made for the benefit of public health and the welfare of farm workers? Ultimately these data may inform Provincial health officials and provide the farm owners/managers a voice to document steps taken to combat risky drinking.

### 1.1. Alcohol Use in South Africa and the Western Cape

SA is the largest country in sub-Saharan Africa in terms of land mass and population [26]. In 1999, the WCP was home to approximately 150,000 farm workers [3]. Deciduous fruit farming accounted for 24% of agricultural production, wine 15%, and wheat 10% [3]. Agriculture remains one of the largest, single employment sectors in SA today, particularly for women [5]. SA is economically dependent on the multi-billion Rand liquor industry [1], and the WCP is the hub for wine production. WCP residents purchase alcoholic beverages from an estimated 5300 licensed liquor outlets and an estimated 25,000 unlicensed/illegal outlets (called shebeens) [11].

In 1961, the annual *per capita* consumption of beverage alcohol in SA was 2.0 L of absolute alcohol (absolute alcohol is a common name for the chemical compound ethanol; ethanol is a colorless liquid with the molecular formula C_2_H_2_OH and it is the alcohol found in alcoholic beverages) [27]. Total liters of absolute alcohol more than doubled from 1961 to 1990 (2.0 to 4.9 L), and beer became five times more popular than wine (52.4 *vs.* 9.3 L). By 2003–2005, the level of annual *per capita* alcohol consumption for adults was estimated at 9.5 L [28].

Drinking to intoxication is common in sub-segments of the SA population where almost a third of male and female current drinkers consume alcohol at risky levels over weekends, with levels being particularly high among drinkers in the Coloured, Black, and non-urban populations [4,13,15,19,22,29]. The terms “White”, “Black”, and “Coloured” originate from the apartheid era, and refer to demographic markers, not inherent characteristics. They refer to people of European, African and mixed (African, European and/or Asian) ancestry, respectively. These designations have historical significance, and their continued use in SA is important for monitoring improvements in health and socio-economic conditions, identifying vulnerable elements of the population, and planning effective prevention programs.

Falletisch [1] studied wine farms from an insider’s perspective and observed the following. Farm laborers are concerned mainly with the present; and consuming alcohol at the end of the day, or more commonly on weekends, is a priority among many [1]. There is anecdotal evidence that habitual drinkers will not buy essential items to save money for alcohol [1]. Some believe that drinking beer does not classify one as a drinker, even when one drinker consumed 12 bottles of beer (750 mL) in one occasion. Some women drink beer because it makes them urinate more; the perception being that a person can drink more before getting drunk [1]. Drinkers in this study believe they have control of their drinking and do not need help to overcome their drinking [1]. Drinking is most commonly a group activity, often serving to unite and promote friendship. There is little to no support to stop drinking among many peer groups [1]. A religious conversion is often reported as linked to successful cessation of drinking [17,19,23]. Among the nine provinces in SA, the WCP has particularly problematic alcohol use [11,30].

In March 2009 owners of two wine farms located in our prevention research community (PC) came to a project office to learn more about FASD studies, and to voice their concerns that the popular press often maligned all wine farmers in the area for continuing to give cheap wine to their workers (e.g., Dop). These farm owners and many others were not pleased to be labeled as contributing to the problem of FASD.

### 1.2. Drinking and Maternal Risk Factors Studies

At the time when the two farm owners came to speak with our staff, we had collected data from 781 women whose children had been screened for an FASD in our developmental clinics in the PC.

We had conducted field research studies in 1997, 1999, 2002, and 2008, and many of the women whose children were diagnosed on the spectrum (the spectrum refers to the severity of the features of FAS when examined by the phyicians on our team. The spectrum may also refer to how well a child performs on the various educational tests. Some children are mildly affected meaning that they are on the low end of the spectrum, whereas others are very severely affected, on the high end of the spectrum) and controls were employed as farm workers. Mothers of children on the spectrum tended to have parents, siblings, friends and partners who drank in riskier patterns, as did the women themselves, when compared to controls. FASD mothers binged (three or more drinks per occasion) more often than controls, and most drinking among all women and men occurred on weekends. Beer and wine were the favorite beverages [17,19,21,24].

## 2. Methods

### 2.1. Community Surveys

Cross-sectional knowledge, attitudes, beliefs, and behaviors (KABB) surveys of the local residents were undertaken independently of any focus on farm characteristics and were part of a large community prevention trial. They were conducted between October 2008 and June 2010 in the PC and four comparison communities (CCs). The community surveys provided a demographic profile of those communities, but more importantly, normative and baseline measures of drinking in those communities.

#### 2.1.1. Design

The community survey questionnaire was comprised of 250 questions and was adapted from various U.S. national household surveys and previous field surveys utilized by members of the study team in the United States and SA. It contained demographic questions, questions on health status and risk behaviors, drinking behavior and associated consequences, questions about use of tobacco and other drugs, and various questions assessing knowledge and attitudes regarding the effects of drinking and the consequences of drinking. Embedded into the questionnaire were two standard problem drinking assessment tools, the World Health Organization’s Alcohol Use Disorders Test (AUDIT) [31] and the CAGE [32]. These tools are validated for use in primary health care and community settings to determine the level of problematic or risky alcohol use, and they provide summary scores useful for comparison. The range of possible scores on the AUDIT is 0–40; scores for the CAGE range from 0–4. A total score of 8 or more on the AUDIT indicates hazardous and harmful alcohol use as well as possible alcohol dependence [31]. The CAGE scale [32] asks if the individual has ever felt that they should cut down on their drinking (C); have been annoyed by being criticized for drinking (A); felt guilty about drinking (G); or have ever had a drink first thing in the morning to steady nerves or get rid of a hangover (E). Individuals with affirmative answers to two or more questions are classified as screening positive for alcohol problems.

#### 2.1.2. Sampling

A cluster random sampling approach was used to select study participants in the community survey. In the PC the predetermined target sample (n = 384) was divided among the nine municipal wards according to the proportion of persons aged 18 to 64 in the 2001 Census [33]. In the CC the target sample comprised 384 participants who were similarly selected from 10 municipal wards. For wards comprising only urban areas, maps of the wards were obtained from the municipality. On each map 4 × 4 centimeter blocks were drawn covering all the wards and each was numbered. A random number generator was then used to select 20% of the blocks per ward. In wards comprising only farms, two persons were interviewed per farm. The number of persons to be interviewed per ward was divided by two, yielding the number of farms that needed to be visited. In wards comprising farms and urban areas, census and other information was used to determine a ratio of residents of farms and urban areas. This ratio was multiplied by the number of interviewees to be selected from the ward to determine the number of residents to be interviewed from farms and urban areas. If a person meeting eligibility criteria was not at home or refused to participate, then interviewers went to a neighboring house (first left, then right, and reversing this the next time they needed to replace someone who was not at a target house) until they obtained someone suitable to interview. Exclusion criteria included persons residing in institutions and persons younger than 18 years and older than 65.

#### 2.1.3. Procedures

Teams of one or two trained interviewers approached potential study participants, explained the study to them, and took them through the consent process. Interviews were conducted in the homes of study participants or outside if necessary to ensure privacy. Respondents were given a Rand 50 (equivalent to $7.15 USD) shopping voucher for completing the survey.

#### 2.1.4. Data Analysis

Based on the literature and previously-collected country-level data concerning the use and abuse of alcohol, our a-priori hypotheses were that males and farm workers (of either gender) would drink at riskier levels than others. More information is contained in Parry, *et al.* [30].

Data were initially processed and analyzed using Epi Info (Version 6) [34], and later analyzed with SPSS Version 20 [35]. Chi-square analyses, Mann-Whitney U, and *t*-tests were used for comparing males and females and farm workers *vs.* non-farm populations for nominal, non-parametric data and for interval level random sample data respectively. For chi-square analyses where the predicted cell values were less than five, Fisher’s exact tests were utilized as indicated by footnotes in Table 1 and Table 2. Also, for the chi-squares, asymptotic corrected significance values (2-sided) were employed via SPSS to adjust for skewness on certain variables. Finally, for *t*-test comparisons which proved to have unequal variances based on Levene’s Test for Equality of Variances, adjusted (2-tailed) significance levels were employed. 

**Table 1:** Demographics and stress by gender and occupation, Western Cape Province, South Africa: 2008–2010.

Variable	All (n = 591)	Males (n = 204)	Females (n = 387)	Test	*p*	Farm Workers (n = 82)	Others (n = 509)	Test	*p*
**Age on day of interview (years) (range 18–64)**									
Mean (SD)	38.0 (12.44)	37.4 (12.98)	38.1 (12.11)	t = −0.651 u = −0.781	0.515 0.435	35.9 (11.98)	38.3 (12.49)	*t* = −1.5 4u = −1.51	0.124 0.131
**Ethnic or racial group ^a^ (%)**									
Black/other	16.6	16.3	16.8			3.6	18.7		
Coloured	62.7	66.5	60.7			96.4	57.4		
White	20.7	17.2	22.5	χ^2^ = 2.50	0.286	0.0	24.0	χ^ 2^ = 46.95	<0.001
**Location of residence (%)**									
Rural	19.6	34.0	12.1			91.6	8.0		
Urban (conventional or informal settlement)	80.4	66.0	87.9	χ^2^ = 40.43	<0.001	8.4	92.0	χ^2^ = 314.46	<0.001
**Years education completed (range 0–14)**									
Mean (SD)	9.6 (3.03)	9.1 (3.52)	9.8 (2.71)	*t* = −2.42 ^b^ u = −1.38	0.016 0.167	6.0 (3.23)	10.1 (2.61)	*t* = −10.86 ^b^ u = −10.37	<0.001 <0.001
**Marital status (%)**									
Single (never married)	31.6	36.1	29.2			24.4	32.6		
Married	45.5	43.1	46.8			28.0	48.1		
Co-habitation	15.4	17.8	14.2			43.9	11.0		
Separated/Divorced/Widowed	7.5	3.0	9.8	χ^2^ = 11.88	0.008	3.7	8.3	χ^2^ = 58.86	<0.001
**Respondent works for money (%)**	52.0	71.1	42.0	χ^2^ = 45.07	<0.001	96.4	44.7	χ^2^ = 76.42	<0.001
**Usual occupation (%)**									
Farm worker	13.9	29.1	5.9			100.0	0.0		
Other occupations	86.1	70.9	94.1	χ^2^ = 59.69	<0.001	0.0	100.0		
**Usual employment status (%)**									
Full time	40.7	64.4	28.2			92.8	32.1		
Part time/seasonal	13.8	7.9	17.0			3.6	15.5		
Unemployed	45.5	27.7	54.8	χ^2^ = 71.70	<0.001	0.0	24.8	χ^2^ = 108.86	<0.001
**If seasonal worker, weeks worked each year (range 2–40)**									
Mean (SD)	18.5 (10.16)	28.0 (9.17)	17.5 (9.88)	*t* = −1.79 u = −1.71	0.083 0.088	24.7 (11.02)	17.8 (10.05)	t = 1.13 u = −1.09	0.265 0.272
**Average weekly income—rand (range 0–7692)**									
Mean (SD)	513.9 (947.49)	724.4 (1177.82)	406.8 (785.68)	*t* = 3.39 ^b^ u = −5.72	<0.001 <0.001	302.4 (124.50)	548.6 (1018.26)	*t* = −5.11 u = −2.81	<0.001 0.005
**Degree to which life is stressful (%)**									
Not stressful/Somewhat	7.0	11.5	4.7			13.3	6.0		
Medium stressful	10.8	14.7	8.9			22.7	9.0		
Very or extremely stressful	82.2	73.8	86.4	χ^2^ = 14.69	0.001	64.0	84.9	χ^2^ = 19.66	<0.001
Mean (SD)	3.7 (0.66)	3.6 (0.81)	3.8 (0.55)	*t* = −3.47 ^b^ u = −3.79	0.001 <0.001	3.5 (0.86)	3.8 (0.61)	*t* = −3.07 u = −4.37	0.003 <0.001
**Causes of stress (% Yes)**									
Alcohol or substance abuse	7.7	6.6	8.3	χ^2^ = 0.46	0.496	16.1	6.5	χ^2^ = 5.76	0.018 ^c^
Marital problems/Relationship	12.3	8.9	13.9	χ^2^ = 2.59	0.11	29.0	10.1	χ^2^ = 17.98	<0.001
Kids/Family	25.5	12.6	32.0	χ^2^ = 22.07	<0.001	19.4	26.5	χ^2^ = 1.46	0.226
Unemployment/Financial	34.7	28.1	37.9	χ^2^ = 4.67	0.031	19.4	36.6	χ^2^ = 7.18	0.007
Neighborhood including: neighbors, gangs, area	2.0	5.4	0.3	χ^2^ = 12.39	<0.001 ^c^	6.5	1.4	χ^2^ = 4.91	0.024 ^c^
Problems associated with anger	2.4	2.4	2.4	χ^2^ = 0.00	1.00 ^c^	9.7	1.6	χ^2^ = 11.21	<0.002 ^c^
Health: HIV/AIDS, sickness, disability	4.6	3.0	5.3	χ^2^ = 1.39	0.237	4.8	4.5	χ^2^ = 0.00	0.753 ^c^
Other issues including gossip, small living space	26.7	30.5	24.8	χ^2^ = 1.90	0.168	27.0	26.5	χ^2^ = 0.06	0.937

Notes: **^a^**: The terms “White”, “Black”, and “Coloured” refer to demographic markers and do not signify inherent characteristics. **^b^**: Values corrected for unequal variances via Levene’s test of equal variance. **^c^**: One or more cells have an expected count <5; Fisher’s exact test utilizing Yates’ continuity correction utilized for chi-square value and significance.

**Table 2:** Alcohol use by gender and occupation, Western Cape Province, South Africa: 2008–2010.

Variable	All (n = 591)	Male (n = 204)	Female (n = 387)	Test	*p*	Farm Workers (n = 82)	All Others (n = 509)	Test	*p*
Drank in lifetime (%) Yes	78.5	91.6	71.4	χ^2^ = 31.77	<0.001	95.1	75.8	χ^2^ = 15.33	<0.001
Age first consumed alcohol Mean (SD)	18.7 (5.03)	17.3 (4.13)	19.6 (5.38)	*t* = −4.92 u = −5.56	<0.001 <0.001	16.8 (3.55)	19.1 (5.19)	*t* = −3.61 u = −4.31	<0.001 <0.001
Age began drinking alcohol regularly Mean (SD)	20.6 (4.88)	19.7 (4.26)	21.3 (5.21)	*t* = −3.21 u = −3.04	0.0010.002	19.7 (3.82)	20.8 (5.08)	*t* = −2.14 u = −1.14	0.034 0.256
Drank in past year (%) Yes	69.6	75.1	65.8	χ^2^ = 4.53	0.033	83.1	66.8	χ^2^ = 8.09	0.004
Among current drinkers, **^a ^**drank in past week (%) Yes	69.3	80.6	60.6	χ^2^ = 14.77	<0.001	84.6	65.5	χ^2^ = 8.92	0.003
Among current drinkers, **^a^** drinks consumed in past week (whole sample) Range 0–99 Mean (SD)	7.1 (11.53)	11.32 (14.91)	3.8 (6.29)	*t* = 5.55 **^b^** u = −5.96	<0.001 <0.001	12.6 (15.24)	5.7 (9.93)	*t* = 3.44 u = −4.59	<0.001 <0.001
Among current drinkers, **^a^** drinks consumed in past week (drinkers only) Range 1–99 Mean (SD)	10.4 (12.67)	14.4 (15.45)	6.3 (7.05)	*t* = 4.96 **^b^** u = −5.46	<0.001 <0.001	15.2 (15.52)	8.9 (11.17)	*t* = 2.75 **^b^** u = −3.65	0.008 <0.001
Among current drinkers **^a^**, binged in past week—(% Yes)	54.2	59.8	48.8	χ^2^ = 2.95	0.086	75.0	47.5	χ^2^ = 13.72	<0.001
Years consumed alcohol Range 1–46 Mean (SD)	16.0 (11.248)	17.0 (12.23)	15.2 (10.83)	*t* = 1.33 **^b^** u = −1.08	0.186 0.282	16.4 (12.06)	15.9 (11.33)	*t* = 0.261 u = −1.06	0.794 0.915
Total AUDIT score Range of scores 0–40 Mean (SD)	5.0 (6.95)	7.9 (8.30)	3.5 (5.52)	*t* = 6.83 u = −7.08	<0.001 <0.001	12.5 (9.42)	3.8 (5.60)	*t* = 7.88 **^b^** u = −8.27	<0.001 <0.001
Audit low-high (%) Low (scores 0–7) High (scores 8–40)	74.4 25.6	57.9 42.1	83.3 16.7	χ^2^ = 44.84	<0.001	32.9 67.1	81.2 18.9	χ^2^ = 86.37	<0.001
Total CAGE score Range of scores 0–4 Mean (SD)	0.9 (1.18)	1.3 (1.31)	0.7 (1.01)	*t* = 4.07 **^b^** u = −3.81	<0.001 <0.001	2.1 (1.11)	0.7 (1.02)	*t = 9.89* u = −8.58	<0.001 <0.001
CAGE low-high (%) Low (total score < 2) High (total score ≥ 2)	64.9 35.1	54.7 45.3	72.8 27.2	χ^2^ = 11.28	0.001	24.6 75.4	74.9 25.1	χ^2^ = 57.34	<0.001
CAGE low-high by ethnic Groups (%) Black Coloured White		Low (<2) 86.2 73.7 98.3	High (≥2) 13.8 26.3 1.7	χ^2^ = 39.46	<0.001				
CAGE low-high by age (Groups %) 18–34 35–44 45+		Low (<2) 74.1 81.2 88.6	High (≥2) 25.9 18.8 11.4	χ^2^ = 14.60	0.001				

Notes: **^a^**: A current drinker is a respondent who has consumed one or more drinks of alcohol in the past year. **^b^**: Values corrected for unequal variances via Levene’s test of equal variance.

### 2.2. Farmer Interviews

The discussion with the two farmers in March, 2009 started us thinking of how we might include a qualitative (farm owner interview) component in our broader study on FASD, because these 11 farms were in our catchment area, and the farm owners were an important social integrative factor for all things happening (e.g., use and abuse of alcohol) on their farms. Our staff had frequent contact with the farm owners, farm owners’ wives, or senior staff in arranging interviews of mothers of children for our in-school studies. We thought that including farmer interviews would compliment the other parts of our work so in early 2011 we planned to complete some farmer interviews. Our focus was threefold: (1) to provide a venue for the farmers to describe working and/or living conditions on those farms; (2) to learn directly whether any of the farms had given Dop to their farm workers; and (3) to learn how life on those farms might be affecting a child’s family environment, the children’s ability to learn, and ultimately the ability of those children to achieve major life ambitions and to become valuable and contributing members of society. A month before we were to start our interviews, in August 2011, the Human Rights Watch (HRW) released its report entitled *South Africa: Ripe with Abuse* [36]; HRW staff had gone onto some farms, gathered some information and wrote a report that was critical of farmers’ treatment of their employees. The timing of the release of the HRW report could not have been worse. Farm owners were now more wary of “strangers” coming on to their farms to gather information. Co-authors Ronel Barnard and Marlene de Vries, the managers of our offices in the PC and CCs, secured appointments for interviews. It would be up to co-authors/interviewers Cudore Snell and Jan Gossage to cement the cooperation of the farm owners.

#### 2.2.1. Design

A convenience sample of farmers via in-person interviews was conducted in the PC and CCs. 

#### 2.2.2. Sampling

The sampling for the farmer interviews was purposeful. The farms were selected in several ways. We selected farms that were involved in different ventures (*i.e.*, livestock, table fruit, wine production), and about half of our sample of farms in the area of the PC and the other half in our CCs. Some of the farms had children who had participated in our studies. With that purposeful approach, individuals on 11 farms were contacted and 11 farms (14 individuals, farm owners, senior managers, and human resources staff) agreed to be interviewed for this sub-study.

#### 2.2.3. Procedures

A semi-structured interviewing process was used in a private setting. All but one of the interviews were completed by authors Cudore Snell and Jan Gossage in both Afrikaans and English; one was completed by author Marlene de Vries (both Cudore Snell and Marlene de Vries are fluent in Afrikaans and English). The authors planned to tape record the interviews, but the HRW report caused unease, and therefore, hand written notes were made during each interview. The interviews focused on four areas: (1) structure of labor on the farms (number of males *vs.* females, number of managers and less skilled employees, working hours, pay); (2) amenities provided by the farmers; (3) the legacy of Dop on these farms, current alcohol use, and risky drinking; and (4) descriptions of those interviewed (age, gender, education, *etc.*). The same outline of open-ended questions was used for all of the 14 interviews, and all interviews were conducted on the respective farms. Author Cudore Snell is a native of this region of the WCP and he initiated the interviews, speaking first in Afrikaans to establish rapport; after Cudore Snell’s introductory comments, the interviews were conducted mostly in English. All of the interviewees mentioned directly or indirectly the HRW report [36] and felt unfairly tarnished by its content. It was clear that the farmers felt that the HRW researchers were too “broad brush” with their findings and as a result they were naturally suspicious of our study and feared criticism. Assurances were provided that information from in the interviews would be objectively reported and remain confidential. This helped to put the farm owners or managers at greater ease. Interviews for nine of the farms were fully completed; one was about 98% complete, and the other one was only about a third complete. It was the opinion of Cudore Snell and Jan Gossage that the content of the HRW report was simply too much of an impediment to obtaining all of the needed information for the one incomplete interview.

#### 2.2.4. Data Analysis

The narrative data were interpreted using content analysis [37,38]. Responses were organized into seven thematic categories (demographics of the persons interviewed; farm description; work force; working conditions; remuneration; amenities; alcohol use) to summarize the responses. Because the sample of farmers/managers was small, computer-assisted analysis was deemed unnecessary, and hand coding was used. Authors Jan Gossage and Cudore Snell analyzed the qualitative data.

## 3. Results

### 3.1. Community Survey Data Profile, Social Conditions and Drinking among Farm Workers and Non-Farm Workers

The sample of individuals from the community surveys is described in Table 1. Two hundred and two respondents were males (34%), and the overall mean age was 38 years (SD = 12.4). Almost two-thirds (64%) of the respondents were Coloured, 80.4% resided in urban neighborhoods, participants completed a mean of 9.6 years of schooling, almost two-thirds of the respondents were married or in co-habitation relationships (67.6%), 13.9% were farm workers, and 40.7 worked full time and 18.5% seasonally. Average weekly income ranged from 0 to 7692 Rands, and the mean was 513.9 Rands (during the time these data were collected, a value of 64 to 43 USD). Eighty-two percent rated their lives as very or extremely stressful, with unemployment and financial hardships being the primary cause of that stress. Table 1 also compares these demographics between males and females and farm workers *vs.* others. More men and farm workers reside in rural settings (*p* < 0.001). Farm workers were: two years younger than others; more likely to be Coloured; much less educated than individuals employed in other occupations (6.0 years of schooling *vs.* 10.1 years) (*p* < 0.001); more likely to be married or co-habituating. Twenty-nine percent of males and 5.9% of females were farm workers (*p* < 0.001), and almost all (96.4%) farm workers were working full time or seasonally (*p* < 0.001). Farm workers’ weekly wages were about half of the wages for others (302.4 Rands *vs.* 548.6 Rands, u = −2.81, *p* = 0.05). Females reported that they were under more stress than males (*p* = 0.001). Overall, more farm workers were of lower socioeconomic status (SES) than others, and males were better off than females.

### 3.2. Alcohol Use

Alcohol consumption measures are presented in Table 2. The analyses reveal that 25 of 28 comparisons were statistically significant by at least one of the three tests of significance employed. Eighty-three percent (68) of farm workers were current drinkers (drank one or more drinks in the past year) as compared to 65.5% (333) of “other” workers that is, persons who were not farm workers (*p* = 0.003). While wine and beer are popular beverages of choice of many respondents, beer is the overall most popular alcoholic beverage; 89.2% (73) of farm workers prefer beer while 67.5% (343) of individuals in other occupations prefer beer (*p* < 0.001). Among current drinkers who consumed alcohol in the week preceding the interview, farm workers consumed almost twice the amount of alcohol when compared to others (15.2 *vs.* 8.9 drinks, *t* = 2.75, *p* = 0.008). Data from these community surveys confirm that bingeing (five or more drinks per occasion for men; three or more drinks for women) is more prevalent among farm workers than others (75.0% *vs.* 47.5% respectively, *p* < 0.001).

Mean AUDIT [31] score for community males was 7.9 (SD = 8.3) and 3.5 (SD = 5.5) for females (*p* < 0.001). The difference of AUDIT scores was even larger when comparing farm workers *vs.* others, 12.5 (SD = 9.4) and 3.8 (SD = 5.6) respectively (*p* < 0.001). The CAGE [32] scale revealed that males had more problematic drinking behaviors than females (45.3% *vs.* 27.2%, *p* = 0.001); 75.4% of farm workers had high (≥2) CAGE scores as compared to 25.1% for individuals in other occupations (*p* < 0.001). CAGE data were also analyzed by ethnic group and by age groupings. Coloured respondents had the highest percentage of high CAGE scores (26.3%) followed by Blacks (13.8%) with 1.7% high scores among Whites (*p* < 0.001). Respondents in the 18–34 age group had the greatest percentage of high CAGE scores (25.9%) *vs.* 18.8% for 35–44 year olds, *vs.* 11.4% for those respondents age 45 or older (11.4%) (*p* = 0.001). 

### 3.3. Results from the Farmer Interviews

Fourteen individuals representing farm ownership interests were asked for and completed interviews; 10 males and four females. Their ages ranged from 29 to 75 (average age = 44.6). Twelve were white and two were Coloured. The group was well educated and had completed 11 to 18 years of schooling (average = 14.76 years). Ten of the individuals’ schooling was related to agriculture; three were specifically trained in wine making. The educational focus of the others was: education; human relations; law; and social work. All but one was married. The religious preference of 12 interviewees was Christian and one chose no specific religion; one individual declined to provide information about religious preference. The length of time the owner or his/her family owned the farm ranged from 16 to 100 years with an average of 41.1 years. Some of the farms were relatively small, some were very large; the workforces ranged in size from 33 to 1340 workers. Among those workers who were permanent hires, they had been a part of the labor force from 1 month to 30 years. One of the interviewees had come to the farm as a young man, promoted over time, and now was a senior manager. Many of the farm managers had considerable pride in the fact that generation after generation of some families had been permanent employees of the farm.

### 3.4. Dop and the Use of Alcohol on These 11 Farms

Four of the farms had never provided Dop to workers, six had given Dop, as was the custom, when previous owners, or earlier generations of family had managed the farm. Information about the use of Dop from one farm was not obtained as that was the very incomplete interview. Three discontinued Dop when the law changed in 1961; one manager discontinued Dop when he took over, and in another case, Dop was discontinued immediately after some of the workers broke into the wine cellar. One owner remarked that it was difficult to wean his workers from Dop. Workers over 50 years in age expected Dop. One farm owner commented that Dop was a factor in poor performance, and when he took over as manager, he added money each week to the pay of those who chose not to consume alcohol. In another instance, when Dop was discontinued, six farm workers left that farm and sought employment where Dop was still provided.

The current farm owners/managers encourage their workers to drink responsibly (*i.e.*, zero tolerance for any alcohol impairment on the job, and no excessive use of alcohol at other times where it would impair judgment or behavior. If a worker resided on the farm, this would mean the worker would respect neighbors by not playing music too loud, or destroying property, or not being abusive toward family members or neighbors). One farm uses a breathalyzer on Mondays if the owner/manager suspected that a worker was intoxicated. Over time on this farm, some farm workers had reported for work intoxicated. From 2000 to 2011 this farm used the breathalyzer 2–3 times a year. In every case the worker was warned verbally. On one farm it was indicated that an intoxicated worker would be dismissed for the day without pay. A written warning was given for the occasional repeat offender. Assistance was given for those seeking treatment for alcohol abuse. (Some of the farms had social workers on staff or on call to help individuals with referrals for alcohol treatment, typically to NGOs (non-governmental organizations)). A few workers who were repeat offenders were subsequently fired. Generally, in recent years, the number of problems related to alcohol use and misuse on these 11 farms were described by the owners/managers as minimal (1–3 incidents per year).

### 3.5. Investing in Human Capital

By reputation and from information shared in the interviews, it was evident that the owners/managers of these farms were concerned about the wellbeing of their workers. Over many years, these 11 farms have provided a wide range of services and benefits to uplift their workers and their families; those amenities are summarized below:

*Adult skills training*: One farm in particular had a very active skills development program to teach reading, writing, character building, alcohol and drug awareness, HIV/AIDS awareness, managing money, healthy food planning, parenting, telephone etiquette, conservation (of water), client service (hospitality), merchandising, stock control, customer care, equipment operation (tractor, forklift, spray equipment), occupational health and safety (fire fighting, first aid, safe handling of chemicals).

*Education and educational facilities*: Many of the farm owners built day care centers (for very young children) or provided financial support for the day care centers. Utilities were provided free of charge, teachers’s pay was often subsidized, and in one instance, an outside educator provided oversight of the day care center to ensure the children were being properly educated. Some farm owners paid all school fees and one provided college scholarships.

*Financial assistance*: Two of the farms lent money to their workers at 0% interest or co-signed loans to buy furniture. The owner of one livestock farm “gives” livestock to its workers, will allow those animals free grazing, and then provide expert assistance in selling the animal at auction.

*Home business*: Several of the farm owners allowed their farm workers to conduct their own business during evenings or weekends; examples were cutting of fire wood, and auto repair. A couple owners gave a portion of the farms land to a group of farm workers and provided some additional financial assistance, after which the group grew and harvested the grapes, processed the grapes with the main farms’ equipment, and then marketed the wine under a separate label.

*Housing*: housing assistance was provided in several ways; one farm owner renovated 15 existing houses when he became the owner, and later built completely new housing for 12 families. Some farmers provided housing on the farm while others provided housing in nearby townships. Utilities (water and electricity) were typically provided at no cost to the farm workers. Some farms have housing committees by which the tenants can voice their concerns. Several farms provided retirement homes.

*New facilities*: In addition to having day care centers, farm owners have constructed sports venues, and after school facilities, community centers, libraries. 

*Incentives for superior performance*: Several farms had “friendly” competitions to encourage excellence in work performance. Cash rewards were given; rewards totaled over 1 million Rand over 9 years at one farm.

*Medical care*: On some farms all medical costs were paid by the owner, or the owner pays medical expenses first and is then reimbursed by the farm worker. On at least one farm, a health professional was readily available.

*Remuneration*: Excepting the one incomplete farm interview; every farm owner or manager took pride in saying their farm paid salaries above or well above the minimum wage. Many farms were ahead of the movement to do so.

*Retirement*: Farm workers were encouraged to save some of their wages and in some instances the farm owners provided a double match. On some farms, after a person has worked for a set period of years, the owners provide retirement pay.

*Social activities*: Provide music lessons, holiday youth programs, women’s clubs.

*Transportation*: Almost every farm owner provided some form of transportation for their farm workers; which included to/from school, church, supermarket, medical appointments, or residences off the farm.

*Other forms of assistance*: work clothing was typically provided. One farmer provided refrigerators so that meats could be kept for longer periods of time without spoiling. Another provided plots of land and seeds for growing vegetables. One farm established a Neighborhood Watch program to help suppress crime.

Authors Cudore Snell and Jan Gossage were invited to tour a day care center, an after school center, and to see some flower and vegetable gardens around some of the farm worker residences. The day care center was spotless, painted with vibrant colors, and the classroom walls were covered with educational materials to stimulate the young children. The after school center was recently constructed. The gardens were beautiful.

## 4. Discussion

The community survey data contrasting farm workers *vs.* others (on the right side of Table 1 and Table 2) are quite compelling, as most demographic, social, and alcohol use analyses are significantly different. More farm workers are low SES and have consumed more alcohol over their lifetime. A higher percentage of farm workers consumed alcohol in the year and week preceding their interview. Among the whole sample of current drinkers, farm workers consumed almost twice the amount of alcohol in the week preceding their interview. Binge drinking is much more common among farm workers and results in more problematic scores on the AUDIT [31] and CAGE [32] questionnaire.

In 2000, the amount of alcohol consumed per adult drinker (~20 L) in SA was among the highest in the world [39], and problematic drinking reported among farmworkers in this study is substantially greater than the national and WCP averages reported by Parry [11] and Peltzer [40]. Problem drinking is particularly acute in WCP and worthy of further analysis to determine key factors driving such high levels of drinking to guide intervention. And, high levels of problem drinking are not confined to the communities researched in this study. McLoughlin and colleagues [41] have reported equally high levels of drinking in other rural areas in the province. The levels of problem drinking in these studies, as measured by CAGE scores ≥2, are roughly 50% higher than were reported in a study of immigrant Latino farmworkers in the Southeastern United States [42].

Farm workers were more likely to live in rural areas, be younger, be Coloured, be less educated, be living in less stable relationships (*i.e.*, with unmarried partner rather than with spouse), earn less weekly pay, and have a lower SES. These demographic and socio-economic features are partial explanations for some of the differences in drinking behavior for farm workers. Other studies conducted in SA have also found problematic drinking to be greater among the less educated [11,40], to be greater among lower SES persons [11,40], and among persons who were Coloured [40]. However, local research has not before shown that problem drinking is greater in rural areas than in the proximal urban areas [11,40] or among younger persons [11,40]. It is likely that it is the greater poverty and stress identified here among some inhabitants of rural areas (particularly farm workers) in the five study areas rather than living in rural areas *per se* that explains the higher levels of problem drinking among farm workers. The legacy of the Dop system has also contributed to problem drinking. London [4] for example, found that workers in the SA deciduous fruit industry with past experience of the Dop system were almost 10 times less likely to be abstainers from alcohol than colleagues without exposure to the Dop system. Norms of drinking formed historically have been translated to the present legacy of heavy recreational drinking on weekends [17,18,19,20,21]. 

## 5. Limitations and the Way Forward

First the information in this paper originates from multiple data sources and methods which are cross-sectional. Exact comparisons made across data sets on relatively similar variables may not be precise. But using multiple data sets and methods adds richness to the breadth and foundational knowledge for the reader to consider. Second, the comparative quantitative data regarding farm workers and others relate to five specific communities in the WCP and may not necessarily be generalizable to all farm workers elsewhere in the province. However, research suggests that the levels of problem drinking are similar in other parts of the WCP [41]. Third, the sample of farm owners and managers was small and based on a convenience sample. Therefore the information obtained might not necessarily be generalizable to all farm owners and managers in the study area and beyond. Fourth, farmworkers on the 11 farms were not interviewed to validate information given by the farm owners/managers. Further research via a survey of farm workers’ perspective would aide understanding of the contextual issues regarding risky drinking in the WCP. Understanding specific socioeconomic and environmental conditions that affect the drinking and culture of drinking among of farm workers, and utilizing larger, more representative studies of the impact of practices of farm owners and managers regarding drinking behavior could be beneficial. Further research is needed to specifically determine whether harmful use of alcohol decreases over time on farms where farm management engages in more proactive practices. The dearth of research on drinking among farm workers in SA and elsewhere makes it difficult to formulate objective and helpful policy.

## 6. Conclusions

The consumption of alcohol among farm workers has a long and controversial history in SA and elsewhere [25]. The drinking behavior of at least one quarter to one half of the farm workers in these communities is clearly problematic and needing intervention.

Since 1997 our research team has worked to accurately describe the maternal and paternal risk factors which impact the possibility that a child will be born with an FASD. In this nested sub-study, we obtained some first-hand insight into the life and working conditions on some of the wine, fruit, and livestock farms in our catchment area. Six out of 10 farm owners and managers had been on farms where the Dop system had been used in the past. They mentioned that many older workers expected to receive the Dop and that it was a factor in poor work performance. The farm owners/managers included in our study are acutely aware that alcohol use and abuse can impact the individuals and families of workers and management alike. These farm owners have made and continue to make improvements to uplift the lives and working conditions of their workers, and to overcome any sense of hopelessness. These owners are engaging their workers to make working conditions and life on their farms better. It is possible that such interventions will be crucial to ameliorating the tragic consequences of drinking that occur in many rural communities in SA. They identified various ways in which they attempted to encourage their workers to: reduce risky drinking through personal, environmental, and policy reforms; drink responsibly; and how they had sought to uplift workers and their families. Such qualitative interview data provide a uniquely rich and grounded perspective that has not been provided before in most studies of drinking behavior in the WCP.
